# Spontaneous Diaphragmatic Rupture in Hypermobile Type Ehlers-Danlos Syndrome

**DOI:** 10.1155/2017/2081725

**Published:** 2017-07-13

**Authors:** Ruchi Amin, Brett H. Waibel

**Affiliations:** ^1^Department of Surgery, East Carolina University, Greenville, NC, USA; ^2^Department of Surgery, University of Nebraska Medical Center, Omaha, NE, USA

## Abstract

Ehlers-Danlos Syndrome refers to a spectrum of connective tissue disorders that have a variety of clinical manifestations. In this case, we present a spontaneous diaphragmatic rupture in a patient with type III Ehlers-Danlos Syndrome. The patient presented with worsening shortness of breath after failure of medical therapy for a presumed pneumonia. A CT scan was obtained which showed diaphragmatic rupture with splenic herniation which was repaired in the operating room via thoracotomy. It is important to include diaphragmatic rupture in the differential diagnosis for patients with connective tissue disease and acute onset tachypnea and pain, as this complication has the potential for significant morbidity without prompt surgical intervention.

## 1. Introduction 

Ehlers-Danlos Syndrome (EDS) refers to a spectrum of inherited connective tissue disorders affecting the skin, joints, and blood vessels. Abnormal collagen formation or deficiency leads to excess joint mobility, cutaneous fragility, and poor wound healing. The hypermobile type (EDS type III) primarily presents with hypermobile joints and mild skin manifestations. We present a case of spontaneous diaphragmatic rupture in a patient with EDS type III.

## 2. Case Description 

**Figure 1:**
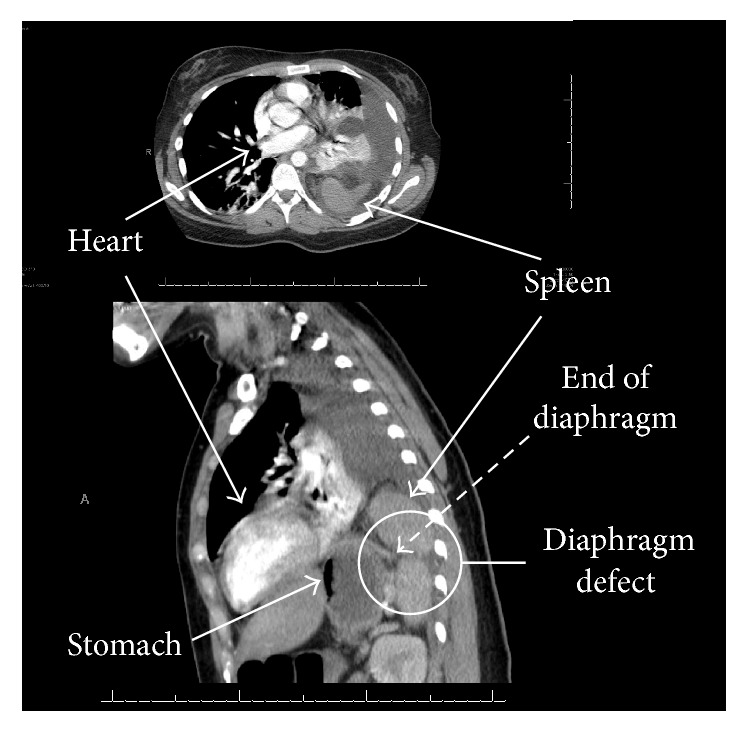
Thoracic CT images showing diaphragm rupture with herniated spleen.

A 27-year-old woman with EDS type III, depression, gastroesophageal reflux, and hypothyroid disease was transferred to our institution with a four-day history of left chest and left upper quadrant abdominal pain. Prior to transfer, she underwent treatment for pneumonia. A thoracentesis for a left parapneumonic effusion was attempted; however, upon obtaining blood, a thoracic CT scan (Figure 1) was performed showing a diaphragmatic rupture with splenic herniation. On arrival, the patient was tachycardic and tachypneic, and she complained of severe left-sided pain with worsening shortness of breath. The patient was taken to the operating room. Upon entering the abdomen, the hernia was visualized with spleen and omentum incarcerating into a 6 × 4 cm defect in the posterolateral left hemidiaphragm. After reduction of the hernia and evacuation of blood in the left hemithorax, the diaphragmatic defect was repaired primarily with large, nonabsorbable (#1 Prolene) interrupted figure of eight sutures. Additionally, a thoracostomy tube was placed to ensure adequate reexpansion of the lung and evacuation of the pleural space. Afterwards, the patient was extubated without difficulty. The patient had an uneventful recovery with gradual improvement in her pain. The thoracostomy tube was removed on postoperative day 5, and she was discharged home the next day.

## 3. Discussion 

EDS is a group of inherited disorders characterized primarily by hyperextensible skin and hypermobile joints, caused by abnormal collagen forms or density. The incidence is estimated at 1 in 5,000 births. Classification is based primarily upon expression of tissue involvement, mode of inheritance, and chemical analysis. Classic (EDS type I, 40%; EDS type II, 40%) and hypermobile (EDS type III, 10%) types comprise approximately 90% of the cases. Classic EDS is characterized principally by skin involvement (hyperextensibility and fragility), with joint hypermobility being more common with the more severe classic form (type I). Hypermobile EDS (type III) has predominantly joint hypermobility with mild to moderate skin involvement and absence of tissue fragility. In type III, a tenascin X deficiency (extracellular matrix glycoprotein that regulates the assembly of collagen fibers) causes reduced collagen density with fragmented elastic fibers. The vascular type (EDS type IV) often has a shortened life span due to life-threatening complications, including sudden death from visceral or great vessel rupture (generally spleen or abdomen viscera) from arterial aneurysm, valvular prolapse, and spontaneous pneumothorax. The other types of EDS are generally more benign, and these individuals rarely present with life-threatening complications [1].

Spontaneous diaphragmatic rupture from strenuous activity (coughing, exercise, vaginal delivery, and vomiting) is a rare surgical emergency. A left-sided rupture is more common, and the defect tends to be located peripherally. Additionally, approximately 25% of cases have a concomitant chest wall defect. These ruptures are associated with visceral organ herniation (in order of frequency): stomach, colon, greater omentum, small intestine, spleen, and liver [2]. Diaphragmatic rupture in our patient was most likely a combination of preexisting weakness associated with her EDS and sudden increase in intra-abdominal pressure due to coughing.

The management of diaphragmatic rupture is surgical, but no widely accepted opinion regarding the optimal approach exists. While some authors advocate performing a thoracotomy for wider exposure of the diaphragm, others advocate a laparotomy, which provides better exposure for management of potential complications (perforation, strangulation, and necrosis of the herniated organs) [2]. The diaphragm is repaired with permanent suture (size 0, #1) in either running or interrupted fashion. In the setting of significant diaphragmatic and/or chest wall destruction, a diaphragm transposition is a suitable alternative [3]. If primary repair is not possible, a nonabsorbable prosthetic mesh (e.g., polytetrafluoroethylene, polyethylene) may be used.

However, in the presence of colonic contamination, the abdomen should be copiously irrigated, and an autologous tissue flap (omentum or latissimus dorsi flap) can be considered [4]. Surgical complication risks with EDS vary depending upon the presentation (classical, hypermobile, and vascular type) but share some common elements. With the collagen defects, all have poor and delayed wound healing with potential for atrophic scar formation. Additionally, the tissue may not hold suture well, especially with joint related surgeries. The vascular type has potential life limiting issues with aneurysm formation and difficulty in repair due to tissue friability. Furthermore, an increased risk in pregnancy related complications, including uterine rupture, has been seen with vascular type EDS. Bleeding disorders are not uncommon due to tissue friability, including easy bruising/mucosal bleeding, despite normal coagulation factors/platelet function.

While this is one of only a handful of cases presented worldwide, it is also the first ever reported case of spontaneous diaphragmatic rupture in patient with EDS type III [5–9]. It is important to include diaphragmatic rupture in the differential diagnosis for patients with connective tissue disease and acute onset tachypnea and pain, as this complication has the potential for significant morbidity without prompt surgical intervention.

## References

[1] Prockop D. J., Bateman J. F., Kasper D., Fauci A., Hauser S., Longo D., Jameson J., Loscalzo J. (2016). Heritable Disorders of Connective Tissue. *Harrison's Principles of Internal Medicine*.

[2] Losanoff J. E., Edelman D. A., Salwen W. A., Basson M. D. (2010). Spontaneous rupture of the diaphragm: Case report and comprehensive review of the world literature. *Journal of Thoracic and Cardiovascular Surgery*.

[3] Bender J. S., Lucas C. E. (1990). Management of close-range shotgun injuries to the chest by diaphragmatic transposition: Case reports. *Journal of Trauma - Injury, Infection and Critical Care*.

[4] Edington H. D., Evans S., Sindelar W. F. (1989). Reconstruction of a functional hemidiaphragm with use of omentum and latissismus dorsi flaps. *Surgery*.

[5] Ratani R. S., Yang D. C., Kalani J. (2000). An intrathoracic wandering spleen in a patient with Ehlers-Danlos syndrome and diaphragmatic hernia. *Clinical Nuclear Medicine*.

[6] Levine M., Adler J. (2011). Acute diaphragmatic rupture in a patient with Ehlers-Danlos syndrome. *Journal of Emergency Medicine*.

[7] Iglesias J. L., Renard T. (1998). Diaphragmatic hernia in an 8-year-old with Ehlers-Danlos syndrome. *Pediatric Surgery International*.

[8] Hamaoui K., Riaz A., Hay A., Botha A. (2012). Massive spontaneous diaphragmatic rupture in Ehlers-Danlos syndrome. *Annals of the Royal College of Surgeons of England*.

[9] Wesley J. R., Mahour G. H., Woolley M. M. (1980). Multiple surgical problems in two patients with Ehlers-Danlos syndrome. *Surgery*.

